# Learning-facilitated synaptic plasticity occurs in the intermediate hippocampus in association with spatial learning

**DOI:** 10.3389/fnsyn.2013.00010

**Published:** 2013-10-29

**Authors:** Jana Kenney, Denise Manahan-Vaughan

**Affiliations:** ^1^Department of Neurophysiology, Medical Faculty, Ruhr University BochumBochum, Germany; ^2^International Graduate School of Neuroscience, Ruhr University BochumBochum, Germany

**Keywords:** LTP, LTD, intermediate hippocampus, dorsoventral axis, *in vivo*, rats, learning, memory

## Abstract

The dorsoventral axis of the hippocampus is differentiated into dorsal, intermediate, and ventral parts. Whereas the dorsal part is believed to specialize in processing spatial information, the ventral may be equipped to process non-spatial information. The precise role of the intermediate hippocampus is unclear, although recent data suggests it is functionally distinct, at least from the dorsal hippocampus. Learning-facilitated synaptic plasticity describes the ability of hippocampal synapses to respond with robust synaptic plasticity (>24 h) when a spatial learning event is coupled with afferent stimulation that would normally not lead to a lasting plasticity response: in the dorsal hippocampus novel space facilitates robust expression of long-term potentiation (LTP), whereas novel spatial content facilitates long-term depression (LTD). We explored whether the intermediate hippocampus engages in this kind of synaptic plasticity in response to novel spatial experience. In freely moving rats, high-frequency stimulation at 200 Hz (3 bursts of 15 stimuli) elicited synaptic potentiation that lasted for at least 4 h. Coupling of this stimulation with the exploration of a novel holeboard resulted in LTP that lasted for over 24 h. Low frequency afferent stimulation (1 Hz, 900 pulses) resulted in short-term depression (STD) that was significantly enhanced and prolonged by exposure to a novel large orientational (landmark) cues, however LTD was not enabled. Exposure to a holeboard that included novel objects in the holeboard holes elicited a transient enhancement of STD of the population spike (PS) but not field EPSP, and also failed to facilitate the expression of LTD. Our data suggest that the intermediate dentate gyrus engages in processing of spatial information, but is functionally distinct to the dorsal dentate gyrus. This may in turn reflect their assumed different roles in synaptic information processing and memory formation.

## Introduction

The dorsoventral axis of the hippocampus is defined into dorsal, intermediate, and ventral parts (Nadel, 1967; Ruth et al., 1982, 1988; Dolorfo and Amaral, 1998; Dong et al., 2009; Fanselow and Dong, 2010). Behavioral studies suggest that whereas the dorsal hippocampus is required for spatial information processing, the ventral hippocampus may be dispensable in this regard (Moser et al., 1993, 1995; Mao and Robinson, 1998; Moser and Moser, 1998; Kjelstrup et al., 2002; Pothuizen et al., 2004). Little is known about the significance of the intermediate hippocampus for information processing and learning.

Two recent studies suggest that it may have a distinct function, however. In a behavioral study, Bast et al. (2009) studied the effect of dorsal, intermediate, and ventral hippocampal lesions on the performance of rats in a Morris water maze task that made high demands on the behavioral flexibility of the animals. Rats with lesions of the intermediate hippocampus were profoundly impaired in rapid place learning. In contrast the ventral hippocampus did not engage in this kind of information processing. Another study compared synaptic plasticity in the dorsal and intermediate dentate gyrus and found that whereas long-term potentiation (LTP) was easier to induce in the intermediate hippocampus, standard afferent stimulation protocols could not elicit long-term depression (LTD); rather short-term depression (STD) that lasted just minutes was evident (Kenney and Manahan-Vaughan, 2013). This finding supports a functional distinction between the intermediate and dorsal hippocampi.

Apart from the widely-reported phenomena of electrically and chemically-induced synaptic plasticity, behaviorally-induced changes in synaptic transmission have also been reported to occur in the hippocampus *in vivo* (Moser et al., 1994; Seidenbecher et al., 1997; Manahan-Vaughan and Braunewell, 1999; Whitlock et al., 2006). A spatial learning event that coincides with a delivery of a stimulation protocol that would normally be subthreshold for induction of long-term plasticity facilitates synaptic plasticity that persists for very long periods (>24 h). This phenomenon is referred to as “learning-facilitated plasticity” (Kemp and Manahan-Vaughan, 2007). The specific forms of facilitated synaptic plasticity depend on the particular hippocampal subregion, as well as on the nature of novel information presented to the animal. Exploration of a novel empty environment facilitates LTP in an input-specific manner at selected synapses in all main hippocampal subregions, whereas learning-dependent facilitation of LTD is subregion specific (Kemp and Manahan-Vaughan, 2004, 2008; Hagena and Manahan-Vaughan, 2011). Namely, in the dorsal dentate gyrus, exploration of large directional cues facilitates persistent LTD, whereas exposure to small, partially hidden cues has no effect on synaptic plasticity. In contrast, in the CA1 region, discrete contextual cues facilitate persistent LTD, whereas large landmark cues do not affect synaptic plasticity (Kemp and Manahan-Vaughan, 2008). Learning-facilitated plasticity is therefore a useful behavioral tool to selectively study functional properties of the hippocampus at the subregional level in freely moving animals.

In this study we examined whether learning-facilitated plasticity occurs in the intermediate dentate gyrus. We observed that whereas learning-facilitated LTP is expressed, learning-facilitated LTD cannot be elicited, at least under the conditions tested here. Rather, we see that STD is enhanced and prolonged, but does not become sustained long enough to support LTD. This finding adds to data that indicate that the intermediate hippocampus may be functionally distinct and serve a specific role in the processing of spatial information in the hippocampus.

## Materials and methods

### Laboratory animals

The present study was carried out in accordance with the European Communities Council Directive of September 22nd, 2010 (2010/63/EU) for care of laboratory animals and after approval of the local ethics committee (Bezirksamt Arnsberg). All efforts were made to minimize animal suffering and to reduce the number of animals. Male Wistar rats (8–12 weeks, Charles River, Germany) were anaesthetized (sodium pentobarbital 60 mg/kg, i.p.) and underwent stereotaxic chronic implantation of bipolar stimulating electrodes in the perforant path (6.9 mm posterior and 4.1 mm lateral from bregma) and a recording electrode in the intermediate granule cell layer of the dentate gyrus (5.9–6.6 mm posterior and 3.9–4.6 lateral from bregma) according to previously described methods (Kenney and Manahan-Vaughan, 2013).

The stimulating and recording electrodes were made of polyurethane-coated stainless steel wire (100 μm diameter; Gündel, BioMedical Intruments, Germany) and were lowered into the brain through a hole drilled on the skull. On the contralateral side, two holes were drilled on the skull into which anchor screws were inserted. The anchor screws were attached to stainless steel wires (A-M Systems, U.S.A.) that served as reference and ground electrodes. The five wires were secured on a six-pin socket (Conrad Electronic SE, Germany) and the whole assembly was stabilized on the skull using dental cement. Test-pulse recordings during surgery aided depth adjustment of the electrodes, which was later verified by postmortem histology. After surgery, rats were housed individually and given at least 7 days recovery time before experiments began. Electrophysiological recordings and behavioral paradigms were performed in 40 (L) × 40 (W) × 60 (H) cm lidless recording chambers wherein (by means of flexible wiring and swivel connectors) the rats could move freely during recordings and had access to food and water *ad libitum*. Animals were transferred in their cages into the experiment room 1 day before the start of experiments to ensure adequate acclimatization to the gross environment.

### Measurement of evoked potentials

The field excitatory postsynaptic potential (fEPSP) was employed as a measure of excitatory synaptic transmission in the DG region. To obtain these measurements, an evoked response was generated in the dentate gyrus by test-pulse stimulation of the perforant path (0.025 Hz) with single biphasic square waves of 0.2 ms duration per half-wave, generated by a constant current isolation unit (World Precision Instruments, U.S.A.). The evoked potential was amplified using a differential AC amplifier (A-M Systems, U.S.A.) and digitalized through a data acquisition unit (Cambridge Electronic Design, U.K.). For each time-point measured during the experiments, 5 consecutively-evoked field responses at 40 s intervals were averaged. The first 6 time-points, which were recorded at 5 min intervals, were averaged and all time points were expressed as a mean percentage (± s.e.m.) of this value. Both the population spike (PS) and the fEPSP were measured. The PS was measured as the maximum of the first negative deflection within the field potential, the fEPSP was quantified by measuring the slope obtained on the second positive deflection of the evoked potential.

Plasticity-inducing electrical stimulation was applied immediately after the sixth time-point and synaptic transmission was recorded for another 4 h (240 min). A further 1 h recording was performed the next day, roughly 24 h after the experiment began, to determine the degree of persistency of any changes in synaptic transmission. By means of an input-output (I/O) curve determination conducted before every experiment, the largest obtainable fEPSP was found for each individual animal (in steps of 100 μA, maximum intensity used 900 μA). The intensity that elicited 40% of the maximum PS (70% in the case of low-frequency stimulation experiments) was used for recordings. Electroencephalography (EEG) activity was monitored throughout the course of the experiment for the occurrence of seizure activity. No behavioral signs or EEG activity indicating seizures were observed.

### Behavioral experiments

In the subsequent experimental phase, novel, or familiar spatial cues were presented to the animals in a familiar environment concurrently with high or low-frequency electrical stimulation. Prior to the beginning of this behavioral experimental phase, each animal underwent control experiments where we, in one experiment, monitored responses to test-pulse stimulation (at 0.025 Hz) for a duration that was equivalent to the recording periods of our plasticity experiment. This was to verify that stable basal synaptic transmission could be evoked in the test animals (Figure 3). In a second experiment we assessed whether the animals respond with synaptic potentiation to high-frequency stimulation (Figure 2) or synaptic depression to low frequency stimulation (Figure 3) in the absence of a behavioral task. This experiment was conducted at least 7 days before the behavioral experiments were commenced. By obtaining an I/O curve at the start of the behavioral experiment (and comparing it to the I/O curve obtained before the control experiments) we verified that no lasting changes in synaptic efficacy had resulted from our control experiments. Only animals that passed this verification were included in behavioral experiments.

**Figure 1:**
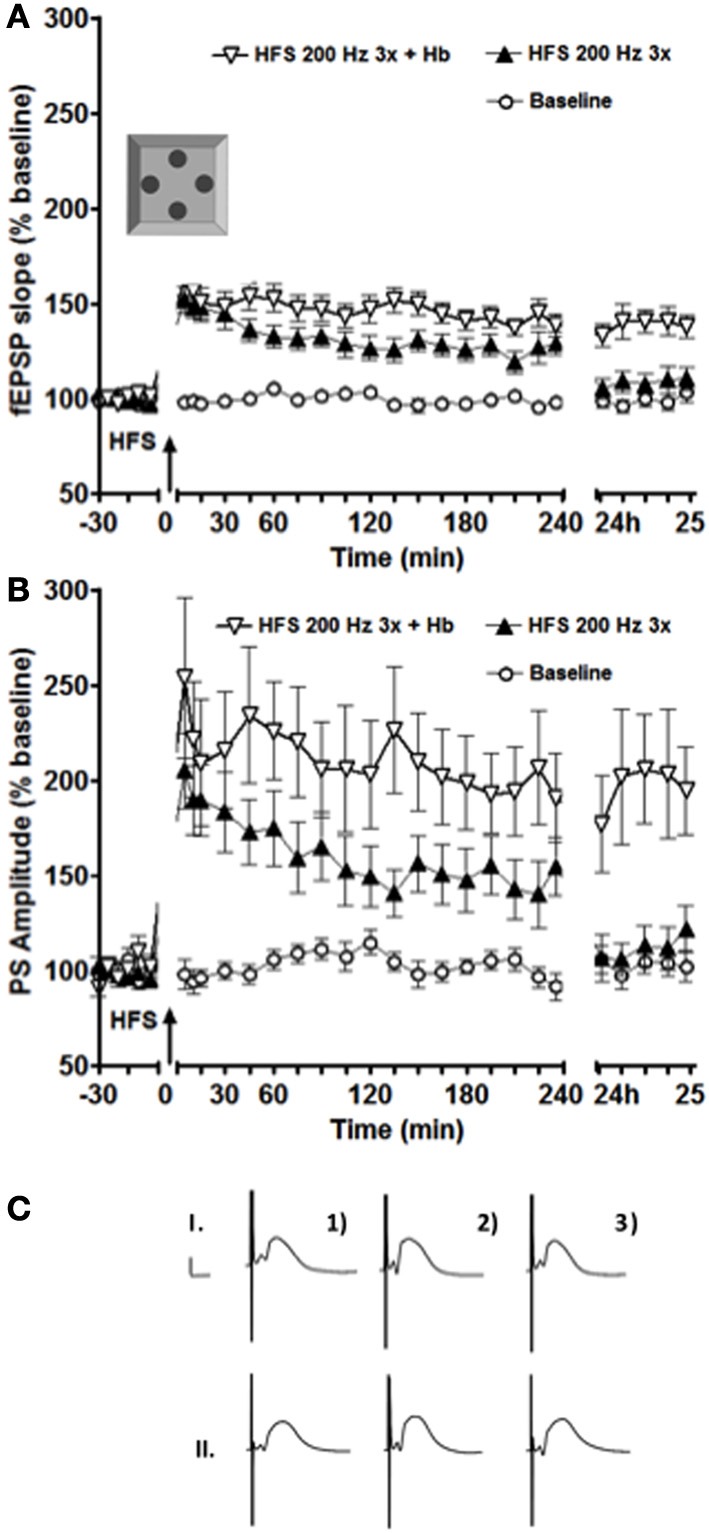
**Exposure to a novel holeboard (Hb) combined with a simultaneous HFS at 200 Hz (3 bursts of 15 stimuli) resulted in a significant facilitation of both fEPSP (A) and PS (B) LTP in the intermediate DG (*n* = 16).** ANOVA revealed a significant difference between the sets of data collected in the experiments with and without holeboard exposure at 24 h after the stimulation onset. **(C)** Shows examples of analog recordings of intermediate DG potentials with (II) and without (I) novel Holeboard exposure 5 min before (1), 5 min after (2), and 24 h after (3) the HFS. Vertical bar: 5 mV, horizontal bar: 5 ms.

To assess if novel space or small contextual features influence synaptic plasticity in the intermediate hippocampus we followed a previously established protocol that was effective in facilitating synaptic plasticity in the hippocampal CA1 region (Manahan-Vaughan and Braunewell, 1999; Kemp and Manahan-Vaughan, 2004) and that consisted of partially concealing small objects in the holes of a holeboard. The holeboard measured 39.8 × 39.8 × 5 cm^2^ (width × length × height) and was made of gray Perspex. The holeboard included four holes of 5.5 cm in diameter that included a cup (gray Perspex) that was 5 cm deep. One hole was located in each quadrant of the holeboard (Figure 1A).

The holeboard was slotted into the recording chamber immediately prior to stimulation. A high-frequency stimulation protocol (200 Hz, 3 bursts of stimuli) was used, as described previously (Kemp and Manahan-Vaughan, 2008). The animals were allowed to explore the holeboard for 15 min before it was removed. The holeboards were washed in soapy water after every use, then thoroughly rinsed and dried.

A re-exposure experiment, whereby the same holeboard was presented to the animals in combination with the same stimulation protocol, was conducted between the 8 and 10th day after the first exposure.

The effect of exposing the rats to novel small objects was examined using the same holeboard as described above. Here, three small identical objects were placed in three of the four holes of the holeboard, as described by Manahan-Vaughan and Braunewell (1999). Low-frequency stimulation (1 Hz, 900 pulses) was applied while the animals were allowed to explore the holeboard and objects for 15 min. After the experiments, the objects were soaked in soapy water overnight, then thoroughly rinsed and dried.

The novel large environmental cues (landmarks) comprised 3 objects of varying height (from 6 to 10 cm) and width/diameter (from 5 to 9 cm) and the objects and procedures used followed those that were previously described as being effective in facilitating LTD in the dorsal dentate gyrus (Kemp and Manahan-Vaughan, 2008). The objects were too heavy for the animals to knock over or move them around. They were made of different materials, so that their surfaces had different textures. The landmarks were placed in three of the four corners of the recording chambers immediately prior to low-frequency stimulation (1 Hz, 900 pulses).

### Data analysis

The results across animals were expressed in terms of mean ± s.e.m. The fEPSPs from the period after electrical stimulation to the end of the experiment were compared. To analyze the electrophysiological data between groups, an analysis of variance (ANOVA) followed by *post-hoc* Fisher LSD test was used. Differences between individual time points were estimated with paired *t*-test. The significance level was set at *p* < 0.05.

## Results

### Exposure to a novel environment (empty holeboard) prolongs LTP in the intermediate dentate gyrus

Exposure to a novel environment (e.g., empty holeboard) facilitates short-term potentiation (STP) into LTP (Kemp and Manahan-Vaughan, 2004, 2008; Hagena and Manahan-Vaughan, 2011). This phenomenon is observed when exploration coincides with high-frequency stimulation that is sub-threshold for LTP induction.

Here, we applied the same stimulation protocol to the intermediate dentate gyrus to find out whether facilitation of LTP also occurs (Figure 1).

This stimulation protocol (200 Hz in 3 bursts of stimuli) elicited LTP in the intermediate dentate gyrus that lasted for over 4 h and less than 24 h. This potentiation was greater than that seen in the dorsal dentate gyrus under identical conditions (Kemp and Manahan-Vaughan, 2008; Kenney and Manahan-Vaughan, 2013). If coupled with exposure to a novel holeboard, LTP was significantly prolonged in the intermediate dentate gyrus, lasting over 24 h [Figure 1, ANOVA: *F*_(1, 30)_ = 13.874, *p* < 0.001 for the fEPSP slope and *F*_(1, 30)_ = 8.2892, *p* < 0.01 for the PS amplitude].

**Figure 2:**
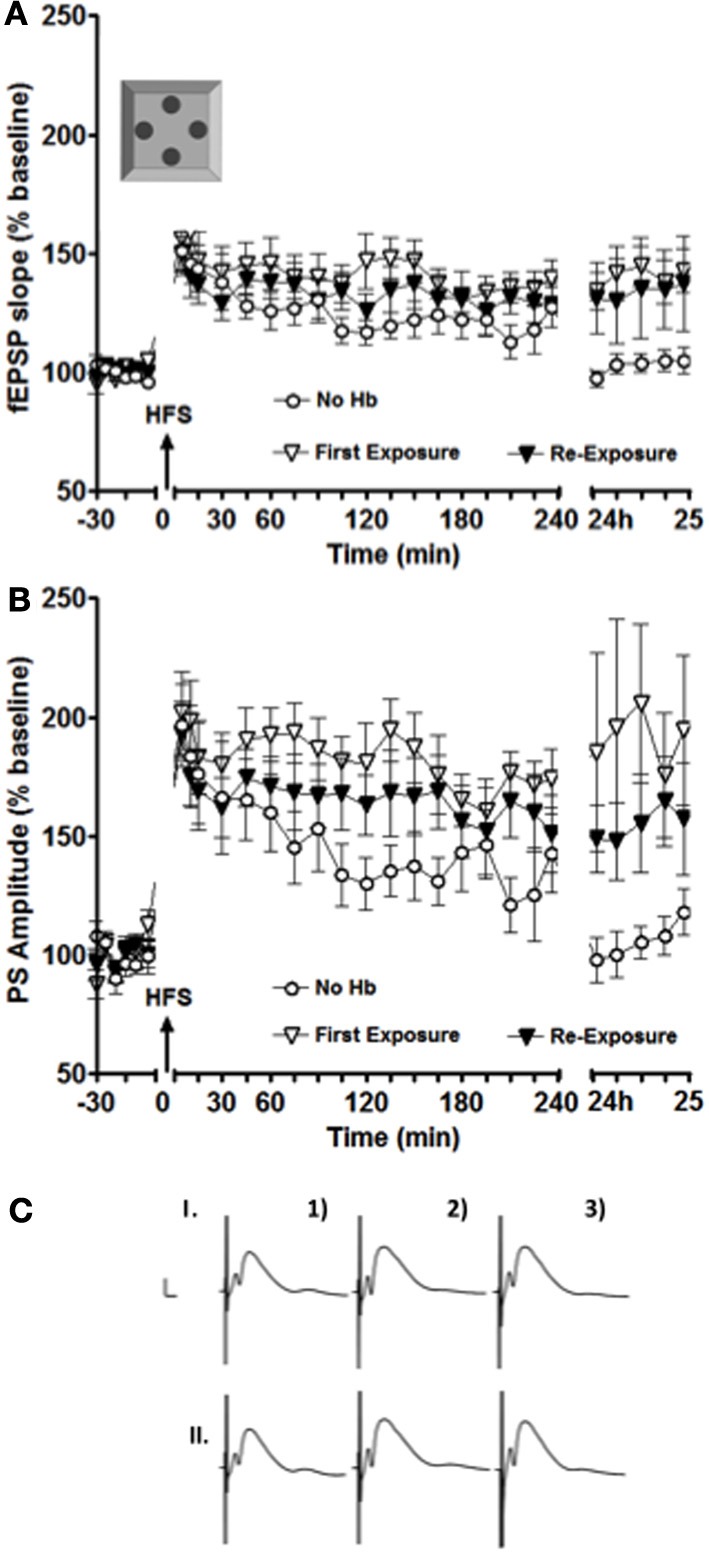
**LTP facilitation was still observed in the intermediate DG after the second exposure to the holeboard. (A,B)** show a facilitation of LTP for both fEPSP **(A)** and PS amplitude **(B)** at 24 h after the first (white inverted triangles) and the second (black inverted triangles) exposure to the holeboard (*n* = 9). The white circle data set represents results from an experiment, where HFS was applied alone without a concurrent holeboard exposure. **(C)** Shows examples of analog recordings of intermediate DG potentials during the first (I) and the second (II) holeboard exposure experiment 5 min before (1), 5 min after (2), and 24 h after (3) HFS. Vertical bar: 5 mV, horizontal bar: 5 ms.

In contrast to previously published results gathered from the dorsal dentate gyrus (Kemp and Manahan-Vaughan, 2008), some degree of facilitation was observed in the present study even in cases when the animals explored the holeboard for the second time and the environment was therefore no longer novel to them (Figure 2).

In this particular experiment, differences were also observed especially on experimental day two (i.e., 24 h after the stimulation onset). Figure 2B shows that, in the case of the PS amplitude, there was a small decrease in the magnitude of LTP facilitation from the first to the second exposure to the empty holeboard. This decrease was, however, not statistically significant [*F*_(1, 16)_ = 0.89719, *p* = 0.36]. Both sets of results were significantly different 24–25 h after high frequency stimulation (HFS) compared to HFS applied alone [ANOVA: *F*_(1, 16)_ = 5.679, *p* < 0.03; *F*_(1, 16)_ = 6.1988, *p* < 0.03, respectively]. The results imply that the second exposure to the holeboard still had some facilitating effect on synaptic plasticity in the intermediate DG (albeit smaller than the first exposure).

### Novel landmark cues enhance STD but do not facilitate LTD in the intermediate dentate gyrus

Exploration of contextual cues facilitates STD into LTD, when novel exploration is coupled with afferent stimulation that in its own right is not sufficient to induced long-lasting synaptic depression (Kemp and Manahan-Vaughan, 2004, 2008). The nature of the cues seems to determine which hippocampal sub-region responds with LTD to novel context exploration. Exploration of small, partially hidden objects facilitates LTD in the Schaffer collateral to (dorsal) CA1 synapse, but not in the perforant path to dorsal DG synapse. On the other hand, dorsal DG, but not dorsal CA1, expresses LTD when afferent stimulation is coupled with the exploration of large landmark cues (Kemp and Manahan-Vaughan, 2004, 2008).

Here, we examined whether the intermediate DG exhibits a similar pattern of responses when the animals are allowed to explore either large or small contextual objects. The exploratory activity of the animals was coupled with a sub-threshold low-frequency stimulation (LFS). The protocol selected as sub-threshold for LTD induction consisted of 900 stimuli delivered at 1 Hz. This particular protocol was selected because it had been already observed to elicit STD when applied in the intermediate DG (Kenney and Manahan-Vaughan, 2013).

**Figure 3:**
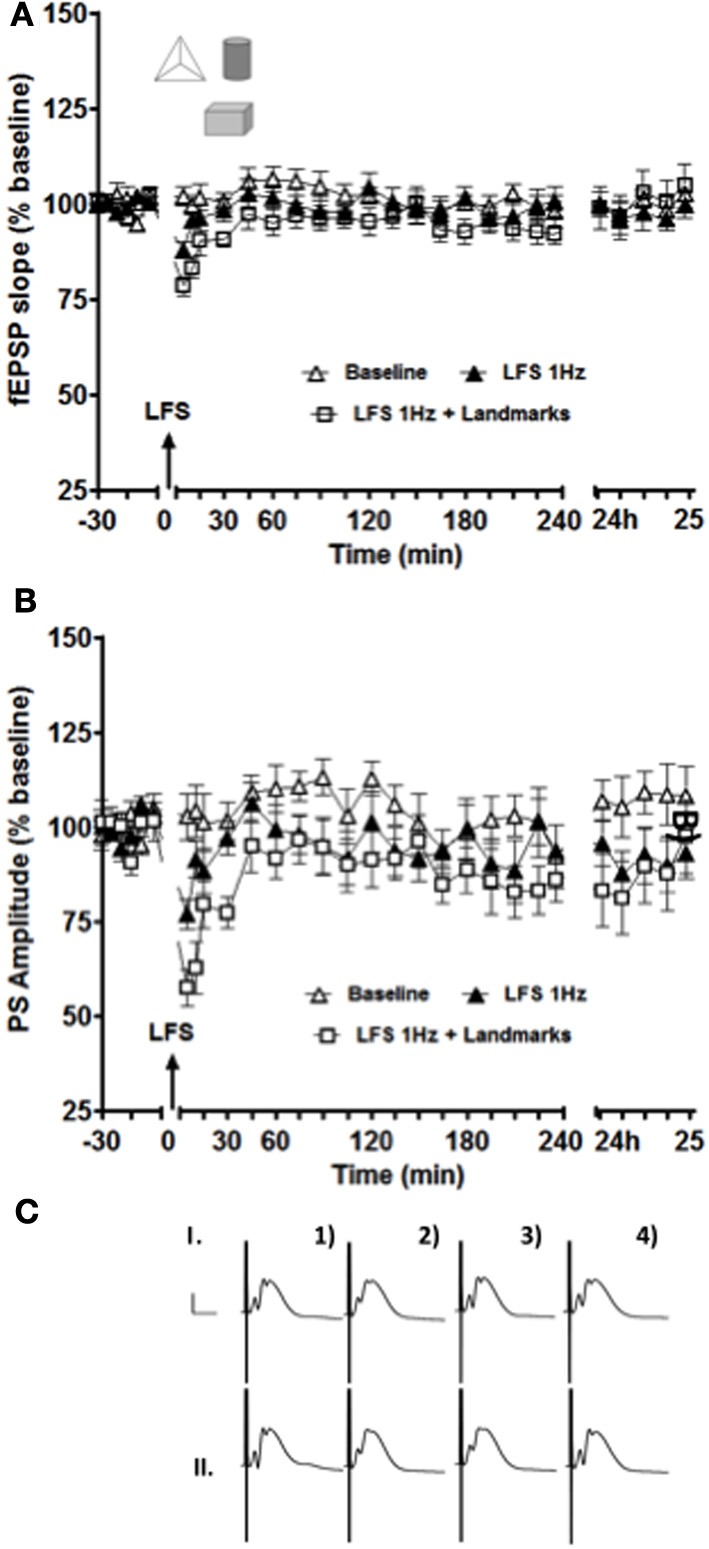
**Short-term depression of both fEPSP (A) and PS (B) amplitude was enhanced and prolonged in the intermediate DG when novel exposure to landmark cues was combined with low-frequency afferent stimulation (white squares data set) compared to low-frequency stimulation alone (black triangle data set).** The white triangle data set represents results collected from the same animals (*n* = 14) in a control test-pulse experiment. **(C)** Shows examples of analog recordings of intermediate DG potentials with (II) and without (I) landmark exposure 5 min before (1), 5 min after (2), 4 h after (3), and 24 h (4) after LFS. Vertical bar: 5 mV, horizontal bar: 5 ms.

Exposure to three novel landmark cues significantly enhanced and prolonged synaptic depression in the intermediate DG when compared to LFS alone (Figure 3). Paired *t*-test revealed significant differences for both fEPSP slope and PS amplitude for the data points collected 5 and 10 min after stimulation (*p* < 0.05 in both cases).

### Exposure to novel small objects enhances STD of the population spike but does not facilitate LTD in the intermediate dentate gyrus

**Figure 4:**
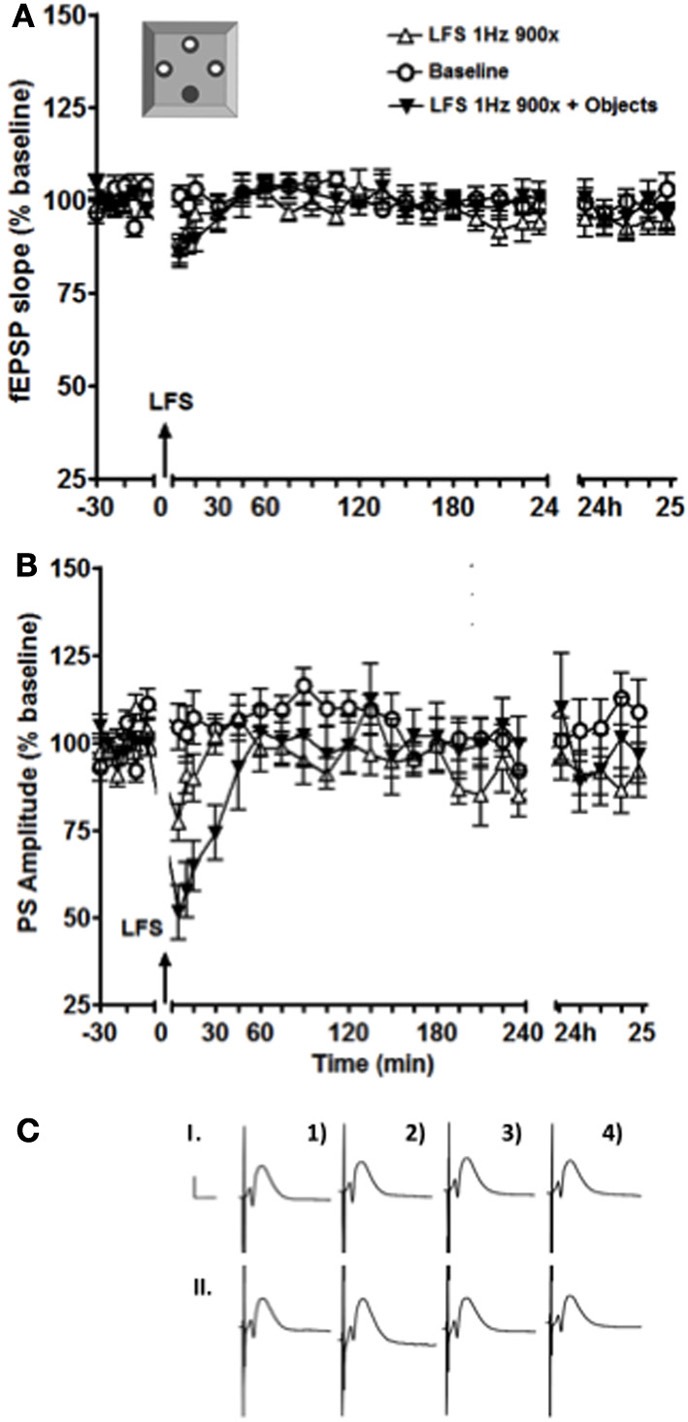
**Exposure to a holeboard containing discrete contextual cues enhanced and prolonged the STD of the PS amplitude (B) but not of the fEPSP slope (A).** The black inverted triangles represent data from an experiment where LFS was applied simultaneously with exposure to the objects, the white triangles represent the results of an experiment where LFS was applied alone, and the white circles show baseline test-pulse responses recorded from the same animals (*n* = 13). **(C)** shows examples of analog recordings of intermediate DG potentials with (II) and without (I) object exposure 5 min before (1), 5 min after (2), 4 h after (3), and 24h (4) after LFS. Vertical bar: 5 mV, horizontal bar: 5 ms.

In the dorsal DG, an exposure to a holeboard containing partially hidden small objects had no effect on synaptic plasticity, although novel landmarks were effective (Kemp and Manahan-Vaughan, 2008). Here we observed no significant effect on the fEPSP of exposure to novel objects in the holeboard holes, whereas an enhancement and prolongation of STD of the PS occurred (Figure 4, *p* < 0.05). The enhancement of STD of the PS persisted for ~30 min before returning back to the baseline levels.

## Discussion

The principal findings of this study suggest that learning-facilitated synaptic plasticity occurs in the intermediate dentate gyrus, however it exhibits differences to responses in the dorsal hippocampus. One the one hand, it shares properties with synapses of the dorsal hippocampus, where it was shown that exposure to novel space facilitates LTP (Manahan-Vaughan and Braunewell, 1999; Kemp and Manahan-Vaughan, 2008; Hagena and Manahan-Vaughan, 2011). On the other hand, in contrast to the dorsal hippocampus, where exposure to novel spatial contextual configurations facilitates persistent LTD (Manahan-Vaughan and Braunewell, 1999; Kemp and Manahan-Vaughan, 2004, 2008; Hagena and Manahan-Vaughan, 2011; Goh and Manahan-Vaughan, 2013), LTD in the intermediate dentate gyrus is not facilitated by this kind of novel spatial experience. Here, only STD was enhanced by novel landmark information, and somatic excitability (population spike amplitude) was transiently reduced by objects that were partially concealed in a holeboard. These data support the hypothesis that the intermediate dentate gyrus may play a role in information coupling that is distinct to that of the dorsal dentate gyrus and dorsal hippocampus.

In the present study, LTP was facilitated in the intermediate dentate gyrus when HFS of the perforant path (that would normally not elicit lasting changes in synaptic strength) was applied concurrently with an exposure to a novel empty holeboard. Intriguingly, this seems to be a common property of all hippocampal synapses studied to date. In the dorsal hippocampus exposure to novel space coupled with subthreshold afferent stimulation facilitates input-specific LTP at the perforant path-dentate gyrus synapse (Kemp and Manahan-Vaughan, 2008), the mossy fiber-CA3 synapse (Hagena and Manahan-Vaughan, 2011), the commissural associational synapse (Hagena and Manahan-Vaughan, 2011), and the Schaffer collateral-CA1 synapse (Manahan-Vaughan and Braunewell, 1999). This phenomenon, that is referred to as learning-facilitated plasticity (Kemp and Manahan-Vaughan, 2007) supports that a very tight link exists between the acquisition and learning of novel spatial experience and the expression of persistent forms of synaptic plasticity in the dorsal hippocampus.

However, a functional differentiation appears to occur with regard to the form of synaptic plasticity expressed during spatial learning. Whereby LTP in the dorsal hippocampus is facilitated by changes in space that could be referred to as being more global, LTD in the dorsal hippocampus is facilitated by changes in spatial content (Kemp and Manahan-Vaughan, 2007; André and Manahan-Vaughan, 2013; Goh and Manahan-Vaughan, 2013). In fact LTD, appears to be more specialized than LTP in this regard. At the level of the perforant path- dentate gyrus synapse and the mossy fiber-CA3 synapse in the dorsal hippocampus, LTD is facilitated by exposure to novel configurations of large objects that are likely perceived as landmarks or navigational cues (Kemp and Manahan-Vaughan, 2008; Hagena and Manahan-Vaughan, 2011). In contrast, at the commissural associational-CA3 synapse and the Schaffer collateral-CA1 synapse, LTD is facilitated by novel exposure to more subtle spatial features such as partially concealed objects or spatially localized odors (Kemp and Manahan-Vaughan, 2004, 2008; André and Manahan-Vaughan, 2013). What is particularly evident is the robustness of the phenomenon in the dorsal hippocampus. This makes it all the more striking that LTD could not be facilitated by either subtle or landmark features in the intermediate hippocampus. Thus, whereas dorsal hippocampal LTD may comprise a cellular mechanism through which the encoding of information about spatial content is enabled, the intermediate hippocampus appears to act distinctly in this regard: exposure to novel landmark cues facilitates transition of STD into LTD in the dorsal dentate gyrus (Kemp and Manahan-Vaughan, 2008), but LTD was never observed in the intermediate hippocampus under identical experimental conditions. Instead, an exposure to novel landmarks markedly enhanced and prolonged STD. This suggests that although the intermediate hippocampus may contribute to spatial information processing, it does not mediate long-term storage of this kind of information.

The lack of LTD response to novel spatial learning may of course be a difference that is restricted to the intermediate dentate gyrus- it has yet to be seen if other synapses in this part of the hippocampal dorsoventral axis also resist facilitation when novel spatial learning occurs, but the absence of learning-facilitated LTD in the intermediate dentate gyrus may indicate that this structure may possess some functional distinctions to the dorsal hippocampus.

For example, whereas the dorsal hippocampus may be involved in the enablement of long-term spatial memory (Kemp and Manahan-Vaughan, 2007), the intermediate hippocampus may play a role in rapid place learning (Bast et al., 2009). This would suggest that the intermediate hippocampus must be equipped to respond and register changes in experience that are then used to modify or update a learned experience. Although we saw facilitation of LTP in the intermediate dentate gyrus following exposure to novel space, this response was different to what was previously observed in the dorsal dentate gyrus and hippocampal synapses as a whole. In the latter case, a very tight correlation exists between the novel learning event and the LTP facilitation (Kemp and Manahan-Vaughan, 2008; Hagena and Manahan-Vaughan, 2011), whereby novel exposure to the spatial environment facilitates LTP but exposure to a learned environment does not. In the intermediate dentate gyrus, LTP was facilitated regardless of whether the spatial environment was novel or not. This renewed facilitation of LTP in the intermediate dentate gyrus that occurred following re-exposure to the now familiar holeboard may reflect a synaptic response that registers a change in the spatial environment or environmental saliency, in line with the postulated role of the intermediate hippocampus in executive control (Burton et al., 2009). The specificity of spatial cues for the facilitation of LTP has not yet been established, thus it is also possible that LTP in the dorsal and intermediate hippocampus are distinct with regard to their sensitivity to different aspects of a spatial environment.

Exposing the animals to novel objects that were placed inside the holeboard holes enhanced STD of the PS (but not fEPSP) and did not lead to LTD. This raises the question as to whether the STD enhancements we observed are more to do with changes in arousal than learning effects. A lack of change of synaptic strength at the level of the field EPSP would indicate that synaptic plasticity did not occur. Rather, changes in somatic excitability took place. Stress enhances LTP in the ventral CA1 (Maggio and Segal, 2007) and noradrenalin release from the locus coeruleus during novelty exploration changes excitability in the dorsal dentate gyrus (Harley, 2007; Sara and Bouret, 2012). Thus it is not unreasonable to assume that arousal factors such as these might affect excitability in the intermediate dentate gyrus. On the other hand, changes in noradrenalin levels in the hippocampus that result from stimulation of the locus coeruleus are tightly linked to the expression of LTD in the dorsal hippocampus (Lemon et al., 2009). Thus, the enhancement of STD of the PS may relate to changes in arousal that are in turn required for effective (short-term) information processing.

## Conclusions

According to Bast et al. (2009) the intermediate hippocampus is critically involved in tasks that are highly demanding at cognitive flexibility. If the intermediate dentate gyrus was to code saliency by means of LTP, then expression of strong and persistent LTP as a reaction to weak stimuli might actually be detrimental to cognitive flexibility. On the other hand, previous data suggest that the threshold for LTP induction may be much lower in the intermediate dentate gyrus compared to the dorsal dentate gyrus (Kenney and Manahan-Vaughan, 2013). This could align with reports that the intermediate hippocampus engages in rapid place encoding (Bast et al., 2009), as this function would require a high sensitivity and synaptic responsiveness to changing information.

The indiscriminate enhancement of STD that we observed is arguably in line with this interpretation. The STD enhancement never persisted for more than 30 min and never became converted into LTD. STD observed in the intermediate DG under these conditions may comprise a mechanism for short-term memory. Its fleeting nature may help the intermediate dentate gyrus to react quickly and flexibly to changing environmental demands, and may act permissively toward the induction of LTP by changing signal-to-noise ratios.

Taken together our results suggest that the intermediate hippocampus is not merely a transitional zone between the dorsal and the ventral poles of the hippocampus, but may subserve a distinct role in spatial information processing.

### Conflict of interest statement

The authors declare that the research was conducted in the absence of any commercial or financial relationships that could be construed as a potential conflict of interest.

## References

[B1] André M. E. Manahan-Vaughan D. (2013). Spatial olfactory learning facilitates long-term depression in the hippocampus. Hippocampus. [Epub ahead of print]. 10.1002/hipo.22158 23804412

[B2] Bast T. Wilson I. A. Witter M. P. Morris R. G. M. (2009). From rapid place learning to behavioral performance: a key role for the intermediate hippocampus. PLoS Biol. 7:e1000089 10.1371/journal.pbio.1000089 19385719 PMC2671558

[B3] Burton B. G. Hok V. Save E. Poucet B. (2009). Lesion of the ventral and intermediate hippocampus abolishes anticipatory activity in the medial prefrontal cortex of the rat. Behav. Brain Res. 199, 222–234 10.1016/j.bbr.2008.11.045 19103227

[B4] Dolorfo C. L. Amaral D. G. (1998). Entorhinal cortex of the rat: topographic organization of the cells of origin of the perforant path projection to the dentate gyrus. J. Comp. Neurol. 398, 25–48 10.1002/(SICI)1096-9861(19980817)398:1<25::AID-CNE3>3.0.co;2-B 9703026

[B5] Dong H. W. Swanson L. W. Chen L. Fanselow M. S. Toga A. W. (2009). Genomic-anatomic evidence for distinct functional domains in hippocampal field CA1. Proc. Natl. Acad. Sci. U.S.A. 106, 11794–11799 10.1073/pnas.0812608106 19561297 PMC2710698

[B6] Fanselow M. S. Dong H. W. (2010). Are the dorsal and ventral hippocampus functionally distinct structures. Neuron 65, 7–19 10.1016/j.neuron.2009.11.031 20152109 PMC2822727

[B7] Goh J. Manahan-Vaughan D. (2013). Spatial object recognition enables endogenous LTD that curtails LTP in the mouse hippocampus. Cereb. Cortex 23, 1118–1125 10.1093/cercor/bhs089 22510536 PMC3615348

[B8] Hagena H. Manahan-Vaughan D. (2011). Learning-facilitated synaptic plasticity at CA3 mossy fiber and commissural-associational synapses reveals different roles in information processing. Cereb. Cortex 21, 2442–2449 10.1093/cercor/bhq271 21493717 PMC3183418

[B9] Harley C. W. (2007). Norepinephrine and the dentate gyrus. Prog. Brain Res. 163, 299–318 10.1016/S0079-6123(07)63018-0 17765726

[B10] Kemp A. Manahan-Vaughan D. (2004). Hippocampal long-term depression and long-term potentiation encode different aspects of novelty acquisition. Proc. Natl. Acad. Sci. U.S.A. 101, 8192–8197 10.1073/pnas.0402650101 15150407 PMC419579

[B11] Kemp A. Manahan-Vaughan D. (2007). Hippocampal long-term depression: master or minion in declarative memory processes. Trends Neurosci. 30, 111–118 10.1016/j.tins.2007.01.002 17234277

[B12] Kemp A. Manahan-Vaughan D. (2008). The hippocampal CA1 region and dentate gyrus differentiate between environmental and spatial feature encoding through long-term depression. Cereb. Cortex 18, 968–977 10.1093/cercor/bhm136 17702951

[B13] Kenney J. Manahan-Vaughan D. (2013). NMDA receptor-dependent synaptic plasticity in dorsal and intermediate hippocampus exhibits distinct frequency-dependent profiles. Neuropharmacology 74, 108–118 10.1016/j.neuropharm.2013.02.017 23499810

[B14] Kjelstrup K. G. Tuvnes F. A. Steffenach H. A. Murison R. Moser E. I. Moser M. B. (2002). Reduced fear expression after lesions of the ventral hippocampus. Proc. Natl. Acad. Sci. U.S.A. 99, 10825–10830 10.1073/pnas.152112399 12149439 PMC125057

[B15] Lemon N. Aydin-Abidin S. Funke K. Manahan-Vaughan D. (2009). Locus coeruleus activation facilitates memory encoding and induces hippocampal LTD that depend on beta-adrenoreceptor. Cereb. Cortex 19, 2827–2837 10.1093/cercor/bhp065 19435710 PMC2774396

[B16] Maggio N. Segal M. (2007). Striking variations in corticosteroid modulation of long-term potentiation along the septotemporal axis of the hippocampus. J. Neurosci. 27, 5757–5765 10.1523/JNEUROSCI.0155-07.2007 17522319 PMC6672761

[B17] Manahan-Vaughan D. Braunewell K. H. (1999). Novelty acquisition is associated with induction of hippocampal long-term depression. Proc. Natl. Acad. Sci. U.S.A. 96, 8739–8744 10.1073/pnas.96.15.8739 10411945 PMC17586

[B18] Mao J. B. Robinson J. K. (1998). Microinjection of GABA-A agonist muscimol into the dorsal but not the ventral hippocampus impairs non-mnemonic measures of delayed non-matching-to-position performance in rats. Brain Res. 784, 139–147 10.1016/S0006-8993(97)01324-3 9518581

[B19] Moser E. Moser M. Andersen P. (1993). Spatial learning impairment parallels the magnitude of dorsal hippocampal lesions but is hardly present following ventral lesions. J. Neurosci. 13, 3916–3925 8366351 10.1523/JNEUROSCI.13-09-03916.1993 PMC6576447

[B20] Moser E. I. Moser M. B. Andersen P. (1994). Potentiation of dentate synapses initiated by exploratory learning in rats: dissociation from brain temperature, motor activity, and arousal. Learn. Mem. 1, 55–73 10467586

[B21] Moser M. B. Moser E. I. (1998). Functional differentiation in the hippocampus. Hippocampus 8, 608–619 10.1002/(SICI)1098-1063(1998)8:6<608::AID-HIPO3>3.0.CO;2-7 9882018

[B22] Moser M. B. Moser E. I. Forrest E. Andersen P. Morris R. G. M. (1995). Spatial learning with a minislab in the dorsal hippocampus. Proc. Natl. Acad. Sci. U.S.A. 92, 9697–9701 10.1073/pnas.92.21.9697 7568200 PMC40869

[B23] Nadel L. (1967). Behavioral Effects of Dorsal and Ventral Hippocampal Lesions in the Rat. Unpublished Ph.D. thesis, Department of Psychology, McGill University, Montreal

[B24] Pothuizen H. H. Zhang W. N. Jongen-Relo A. L. Feldon J. Yee B. K. (2004). Dissociation of function between the dorsal and the ventral hippocampus in spatial learning abilities of the rat: a within-subject, within-task comparison of reference and working spatial memory. Eur. J. Neurosci. 19, 705–712 10.1111/j.0953-816X.2004.03170.x 14984421

[B25] Ruth R. E. Collier T. J. Routtenberg A. (1982). Topography between the entorhinal cortex and the dentate septotemporal axis in rats: I. Medial and intermediate entorhinal projecting cells. J. Comp. Neurol. 209, 69–78 10.1002/cne.902090107 7119174

[B26] Ruth R. E. Collier T. J. Routtenberg A. (1988). Topographical relationship between the entorhinal cortex and the septotemporal axis of the dentate gyrus in rats: II. Cells projecting from lateral entorhinal subdivisions. J. Comp. Neurol. 270, 506–516 10.1002/cne.902700404 2836479

[B27] Sara S. J. Bouret S. (2012). Orienting and reorienting: the locus coeruleus mediates cognition through arousal. Neuron 76, 130–141 10.1016/j.neuron.2012.09.011 23040811

[B28] Seidenbecher T. Reymann K. G. Balschun D. (1997). A post-tetanic time window for the reinforcement of long-term potentiation by appetitive and aversive stimuli. Proc. Natl. Acad. Sci. U.S.A. 94, 1494–1499 10.1073/pnas.94.4.1494 9037081 PMC19819

[B29] Whitlock J. R. Heynen A. J. Shuler M. G. Bear M. F. (2006). Learning induces long-term potentiation in the hippocampus. Science 313, 1093–1097 10.1126/science.1128134 16931756

